# Polymorphism rs189037C > T in the promoter region of the ATM gene may associate with reduced risk of T2DM in older adults in China: a case control study

**DOI:** 10.1186/s12881-017-0446-z

**Published:** 2017-08-14

**Authors:** Xiang Ding, Qiukui Hao, Ming Yang, Tie Chen, Shanping Chen, Jirong Yue, Sean X. Leng, Birong Dong

**Affiliations:** 1 Center of Gerontology and Geriatrics, West China Hospital, Sichuan University, NO. 37, Guoxuexiang, Chengdu, Sichuan 610041 China; 2 Institute of Molecular Medicine, Health Science Center, Shenzhen University, Shenzhen, 518060 China; 3 Division of Geriatric Medicine and Gerontology, Department of Medicine, Johns Hopkins University School of Medicine, 5501 Hopkins Bayview Circle, Baltimore, MD 21224 USA

**Keywords:** Ataxia telangiectasia mutated, rs189037, Single nucleoside polymorphism, Type 2 diabetes mellitus, Coronary artery disease

## Abstract

**Background:**

Recent evidence indicates that ataxia telangiectasia mutated (ATM) is a cytoplasmic protein that involves in insulin signaling pathways. When ATM gene is mutated, this event appears to contribute to the development of insulin resistance and type 2 diabetes mellitus (T2DM). Up to date, little information about the relationship between ATM gene polymorphism and T2DM is available. This study aimed to explore potential association between a genetic variant [single nucleotide polymorphism (SNP), i.e. rs189037C > T] in the ATM promoter region and T2DM in older adults in China.

**Methods:**

We conducted a 1:1 age- and sex-matched case-control study. It enrolled 160 patients including 80 type 2 diabetic and 80 nondiabetic patients who were aged 60 years and above. Genotyping of the polymorphism rs189037 in the promoter of the ATM gene was performed using polymerase chain reaction-restriction fragment length polymorphism. Chi-square test or Fisher’s exact test (when an expected cell count was <5) and unpaired Student’s *t* test were used for categorical and continuous variables, respectively. Logistic regression was used to estimate odds ratio (OR) and 95% confidence interval (CI) with adjustment for factors associated with T2DM.

**Results:**

Significant association was found between the genotypes of the ATM rs189037 polymorphism and T2DM (*P* = 0.037). The frequency of CT genotype is much higher in patients without T2DM than in diabetics (60.0% versus 40.0%, *P* = 0.012). After adjustment of the major confounding factors, such difference remained significant (OR for non-T2DM is 2.62, 95%CI = 1.05–6.53, *P* = 0.038). Similar effect of CT genotype on T2DM was observed in male population (adjusted: OR = 0.27, 95%CI = 0.09–0.84, *P* = 0.024). In addition, the percentage of TT genotype in diabetics with coronary artery disease (CAD) was considerably lower than in those without CAD (17.9% versus 61.5%, *P* = 0.004).

**Conclusions:**

Our study suggests that the ATM rs189037 polymorphism is associated with reduced risk of T2DM in older adult population in China. Specifically, CT heterozygote seems to be associated with a lower risk of T2DM than CC or TT genotype, especially in male older adults. Moreover, TT genotype may reduce the risk of CAD in diabetic patients.

**Electronic supplementary material:**

The online version of this article (doi:10.1186/s12881-017-0446-z) contains supplementary material, which is available to authorized users.

## Background

The prevalence of type 2 diabetes mellitus (T2DM) has increased dramatically. It represents a primary challenge to health care and is considered as a major international health concern with great impact on global morbidity, premature mortality, and related complications, especially in old adults [1, 2]. The International Diabetes Federation in 2013 estimates that about 382 million people have diabetes, and the number will expand to 592 million in the next 25 years worldwide. Diabetes will be the 7th leading cause of death [3, 4]. Substantial evidence suggests that T2DM is a complex multifactorial disease resulting from interactions between genetic and environmental factors [5, 6]. Ataxia telangiectasia mutated (ATM) gene, first cloned by Savitsky et al. in 1995, is located on human chromosome 11q22–23 [7]. The gene product, ATM protein, is a 370 kDa serine/threonine protein kinase that contains 3056 amino acids residues and a member of phosphoinositide 3-kinase-related protein kinase family [8, 9]. When functional ATM protein is deficient, this event may lead to a rare autosomal recessive genetic disease - ataxia-telangiectasia (AT). The most prominent characteristics of AT are neurodegeneration, oculocutaneous telangiectasias, genomic instability, a high risk for cancer, immune deficiencies, growth retardation, and premature aging [8, 10–13]. AT patients also display a predisposition to glucose transporter aberrations, insulin resistance and glucose intolerance, all of which are important features of T2DM [14, 15]. Previous study has demonstrated that ATM kinase is a nuclear protein and plays a critical role in the response to DNA double strand breaks by phosphorylating a large number of downstream substrates that are involved in DNA repair, cell arrest, chromatin remodeling and apoptosis [16]. Several recent studies have also shown that ATM is a cytoplasmic protein that participates in many cytoplasmic processes that influence cellular homeostasis and metabolism, especially in insulin signaling pathways [10, 17, 18]. The effect of ATM on the insulin signaling is mainly mediated by phosphorylation of the serine-threonine kinase Akt [18, 19] and p53-dependent pathway [20]. Decreased Akt and p53 phosphorylation due to ATM gene mutations contribute to the development of insulin resistance and T2DM, which suggests that the lack or inactivation of ATM protein might play a role in the pathogenesis of T2DM. Moreover, Schneider et al. observed that ATM-deficient mice were prone to increase vascular disease and insulin resistance [19].

A number of studies have reported associations of single nucleotide polymorphisms (SNPs) of the ATM gene with increased risk for several cancers, such as breast cancer, lung cancer, thyroid carcinoma, pancreatic cancer and nasopharyngeal carcinoma [21–26]. Whether the SNPs of ATM gene are associated with the risk of T2DM remains unknown. The objective of this study was to explore potential relationship between the rs189037 polymorphism, one of the SNPs of ATM gene, and T2DM among older adults in China, which may provide new insights into the genetic mechanisms of T2DM.

## Methods

### Subjects

A total of 160 patients aged 60 years or over, 80 type 2 diabetics and 80 age- and sex-matched nondiabetics, were recruited from the department of Geriatrics at West China Hospital, Sichuan University (Chengdu, China) between September 2012 and July 2013. T2DM was diagnosed according to the World Health Organization criteria [27] or had a documented clinical diagnosis of T2DM from medical records. Patients with other types of diabetes (i.e., type 1 diabetes and gestational diabetes) or malignant tumors were excluded. Control subjects had no history of diabetes mellitus or cancer. Their fasting glucose values were below 5.6 mmol/L without taking any glucose-lowering medication. All participants were unrelated Chinese and underwent biochemical testing. The study was in accordance with the principles of the Declaration of Helsinki and was approved by the clinical trials and biomedical ethics committee of West China Hospital, Sichuan University. Written informed consent for participation in the study and the donation of samples were obtained from all participants and their legal proxies.

### Genotyping

Genomic DNA was extracted from EDTA-treated whole blood samples collected from each patient with Blood Genomic extraction kits according to the manufacturer’s instruction (DP319, TianGen, Beijing, China). SNP rs189037 of the ATM gene was genotyped using polymerase chain reaction restriction fragment length polymorphism (PCR-RFLP). Forward primer 5′-GCTGCTTGGCGTTGCTT-3′ and reverse primer 5′-CATGCGATTGGCGGTCTGG-3′ (TaKaRa, Dalian, China) were designed and synthesized as described in our previous study [28]. The amplification conditions were used as follows: initial denaturation at 94 °C for 3 min, followed by 30 cycles of amplification which included denaturation at 94 °C for 30 s, annealing at 55 °C for 30 s, and extension at 72 °C for 30 s with a final extension of 5 min at 72 °C. The PCR products were digested with SacII restriction enzyme according to the manufacturer’s instructions (TaKaRa, Dalian, China). The digestion products were resolved by 10% polyacrylamide gel electrophoresis and stained with silver nitrate. TT homozygous genotype was marked by 125 bp and 162 bp fragments; CC genotype was marked by three fragments of 46 bp, 116 bp and 125 bp; and CT heterozygous genotype by four fragments of 46 bp, 116 bp, 125 bp and 162 bp.

### Assessment of covariates

Baseline characteristics were collected from all participants, including fasting plasma glucose (FPG, mmol/L), uric acid (UA, mmol/L), triglycerides (TG, mmol/L), total cholesterol (TC, mmol/L), low-density lipoprotein cholesterol (LDL-C, mmol/L), high-density lipoprotein cholesterol (HDL-C, mmol/L), smoking habits, and chronic diseases containing hypertension and coronary artery disease (CAD). FPG, UA, and lipid/lipoprotein levels were determined using standard laboratory techniques. The diagnosis of hypertension was made if patients were under treatment or the mean blood pressure of 3 measurements was >140/90 mmHg. CAD was diagnosed based on coronary angiography as well as clinical criteria.

### Statistical analyses

All statistical analyses were performed using SPSS software (version 19.0, SPSS, Inc., Chicago, IL, USA). The Hardy–Weinberg equilibrium was carried out by a chi-square test. Chi-square tests or Fisher’s exact tests (when an expected cell count was <5) were used for categorical variables, and unpaired Student’s *t* tests were used for continuous variables. The genotypes and alleles frequencies between the case and control groups were compared using chi-square test. Unconditional multiple logistic regression analyses were employed to estimate unadjusted and adjusted odds ratios (ORs) and 95% confidence intervals (CIs). Adjusted ORs and *P* values were corrected for factors associated with T2DM that included sex, age, FPG, UA, blood lipid levels, and smoking status, CAD and hypertension. Statistical significance was determined by a *P* value <0.05. All *P* values were two-sided.

## Results

Among 160 participants, 112 males and 48 females, the mean age was 69.5 ± 5.7, ranging from 60 to 81 years. Table 1 shows the clinical characteristics of patients with and without T2DM. Patients with T2DM had significantly higher FPG and lower HDL-C levels than controls. The prevalence of CAD or hypertension was significantly higher in the patient group than that in the control group. UA, TG, TC, LDL-C, and smoking did not differ significantly between the two study groups (Additional file 1).

**Table 1:** Clinical characteristics of the study patients according to T2DM

Variables	T2DM group (*n* = 80)	Control group (*n* = 80)	Wilcoxon W or χ^2^	*P* value
FPG (mmol/L)	8.04 ± 3.34	5.29 ± 1.60	4272.5	0.000^*^
UA (mmol/L)	328.02 ± 98.48	346.02 ± 100.18	5779.5	0.508
TG (mmol/L)	1.59 ± 0.66	1.56 ± 0.72	5960.0	0.465
TC (mmol/L)	3.92 ± 1.11	4.17 ± 1.14	5283.0	0.217
LDL-C (mmol/L)	2.29 ± 0.83	2.39 ± 0.82	5441.0	0.511
HDL-C (mmol/L)	1.11 ± 0.27	1.24 ± 0.33	4870.5	0.006^*^
Smoking, n (%)	45 (56.3)	41 (51.3)	0.40	0.526
CAD, n (%)	67 (83.8)	54 (67.5)	5.73	0.017^*^
Hypertension, n (%)	54 (67.5)	39 (48.8)	5.78	0.016^*^

*T2DM* Type 2 diabetes mellitus, *FPG* Fasting plasma glucose, *UA* Uric acid, *TG* Triglycerides, *TC* Total cholesterol, *LDL-C* Low-density lipoprotein cholesterol, *HDL-C* High-density lipoprotein cholesterol, *CAD* Coronary artery disease

^*^: *P* < 0.05

Genotype frequencies of rs189037 polymorphism in the ATM gene promoter region were detected to conform to the Hardy-Weinberg equilibrium in the case and control groups (case group: *P* = 0.195; control group: *P* = 0.202. Additional file 2: Table S1). Table 2 shows genotype distribution of the rs189037 SNP and its associations with T2DM in the study population. Among patients with T2DM, CC genotype was found in 28 (35.0%), CT in 32 (40.0%), and TT in 20 (25.0%) subjects. The frequency of the C allele in the case group was 55.0%. Among the age- and sex-matched non-diabetic controls, CC genotype was observed in 17 (21.3%), CT in 48 (60.0%), TT in 15 (18.7%), and the C allele in 41 (51.3%) subjects. A comparison of genotypic frequencies revealed that there was a significant association between SNP rs189037 polymorphism and T2DM (*P* = 0.037). For example, the non-diabetic controls exhibited a higher frequency of the CT genotype than the diabetic patients (60.0% versus 40.0%, *P* = 0.012). Compared to the CC/TT genotypes, a significantly lower risk of T2DM was observed in the subjects with CT heterozygous genotype with OR = 2.62, 95%CI = 1.05–6.53, *P* = 0.038, adjusted for factors associated with T2DM t including FPG, UA, blood lipid levels, and histories of smoking, CAD and hypertension. Sex stratification analysis revealed significant association between the ATM rs189037 polymorphism and T2DM (*P* = 0.045) among male participants. The CT genotype exhibited significant protection against T2DM (62.5% among male controls versus 41.1% among male diabetics, *P* = 0.024; adjusted OR = 0.27, 95%CI = 0.09–0.84, *P* = 0.024) (Table 3). Due to limited sample size (only 48 females included in the study), we precluded similar analysis in female participants.

**Table 2:** Genotypes distributions of ATM rs189037 and the association with T2DM (*n* = 160)

	T2DM group (*n* = 80)	Control group (*n* = 80)	χ^2^	*P* value
Genotypes, n (%)				
CC	28 (35.0)	17 (21.3)	6.60	0.037^*^
CT	32 (40.0)	48 (60.0)		
TT	20 (25.0)	15 (18.7)		
C allele frequency, %	55.0	51.3	0.45	0.501
	OR (95%CI)		*P* value	
CC versus others^a^				
Unadjusted	0.50 (0.25–1.02)		0.055	
Adjusted	1.61 (0.62–4.18)		0.332	
CT versus others^b^				
Unadjusted	0.44 (0.24–0.84)		0.012^*^	
Adjusted	2.62 (1.05–6.53)		0.038^*^	
TT versus others^c^				
Unadjusted	1.44 (0.68–3.08)		0.340	
Adjusted	0.46 (0.16–1.31)		0.146	

^a^: CT and TT; ^b^: CC and TT; ^c^: CC and CT. *OR* Odds ratio, *CI* Confidence interval. *T2DM* Type 2 diabetes mellitus, *FPG* Fasting plasma glucose, *UA* Uric acid, *TG* Triglycerides, *TC* Total cholesterol, *LDL-C* Low-density lipoprotein cholesterol, *HDL-C* High-density lipoprotein cholesterol, *CAD* Coronary artery disease

^*^: *P* < 0.05

**Table 3:** Genotype distributions of the ATM rs189037 and the association with T2DM in males (*n* = 112)

	T2DM group (*n* = 56)	Control group (*n* = 56)	χ^2^	*P* value
Genotypes, n (%)				
CC	22 (39.3)	11 (19.6)	6.20	0.045^*^
CT	23 (41.1)	35 (62.5)		
TT	11 (19.6)	10 (17.9)		
Allele, %				
C	59.8	50.9	1.81	0.179
	OR (95%CI)		*P* value	
CC versus others^a^				
Unadjusted	0.38 (0.16–0.88)		0.025^*^	
Adjusted	2.78 (0.88–8.79)		0.082	
CT versus others^b^				
Unadjusted	0.42 (0.20–0.89)		0.024^*^	
Adjusted	0.27 (0.09–0.84)		0.024^*^	
TT versus others^c^				
Unadjusted	1.12 (0.44–2.91)		0.809	
Adjusted	1.87 (0.48–7.25)		0.368	

^a^: CT and TT; ^b^: CC and TT; ^c^: CC and CT. *OR* Odds ratio, *CI* Confidence interval. *T2DM* Type 2 diabetes mellitus, *FPG* Fasting plasma glucose, *UA* Uric acid, *TG* Triglycerides, *TC* Total cholesterol, *LDL-C* Low-density lipoprotein cholesterol, *HDL-C* High-density lipoprotein cholesterol, *CAD* Coronary artery disease

*: *P* < 0.05

The genotypes of ATM rs189037 polymorphism were tested for interactions with major risk factors of diabetes mellitus, such as CAD, hypertension, smoking, UA and lipid profiles. Table 4 shows different genotype group characteristics. FPG level was significantly lower in the CT genotype than that in the CC or TT genotype group (*P* = 0.049). UA, TG, TC, LDL-C, HDL-C, history of smoking, CAD and hypertension were not significantly different among the CC, CT and TT genotype groups. Among the diabetics, a significant difference in the rs189037 polymorphism was found between the patients with and without CAD (genotypes: *P* = 0.004; alleles: *P* = 0.002). And the frequency of TT genotype in the T2DM patients with CAD was much lower than in those without CAD (17.9% versus 61.5%). As the number of control group is very small (*n* = 13), we did not perform these logistic regression analysis (Additional file 2: Table S2).

**Table 4:** Clinical characteristics of the study patients according to the rs189037 polymorphism in the ATM gene

Variables	CC (*n* = 45)	CT (*n* = 80)	TT (*n* = 35)	χ^2^	*P* value
FPG (mmol/L)	7.67 ± 3.41	6.05 ± 2.24	6.43 ± 3.22	6.03	0.049^*^
UA (mmol/L)	333.29 ± 83.60	340.02 ± 106.27	336.32 ± 105.30	0.01	0.997
TG (mmol/L)	1.56 ± 0.64	1.61 ± 0.74	1.48 ± 0.63	0.43	0.805
TC (mmol/L)	3.91 ± 1.18	4.12 ± 1.13	4.07 ± 1.09	1.33	0.514
LDL-C (mmol/L)	2.28 ± 0.74	2.32 ± 0.83	2.47 ± 0.91	1.26	0.532
HDL-C (mmol/L)	1.17 ± 0.29	1.17 ± 0.33	1.20 ± 0.29	0.31	0.857
Smoking, n (%)	23 (51.1)	48 (60)	15 (42.9)	3.05	0.217
CAD, n (%)	38 (84.4)	61 (76.3)	22 (62.9)	5.01	0.082
Hypertension, n (%)	24 (53.3)	48 (60)	21 (60)	0.59	0.744

*FPG* Fasting plasma glucose, *UA* Uric acid, *TG* Triglycerides, *TC* Total cholesterol, *LDL-C* Low-density lipoprotein cholesterol, *HDL-C* High-density lipoprotein cholesterol, *CAD* Coronary artery disease

^*^: *P* < 0.05

## Discussion

Although many studies have shown that symptoms of insulin resistance, glucose intolerance and T2DM are more frequently observed in AT patients than in the general population, few studies explored its underlying mechanisms. Schalch et al. [29] observed that 59% of AT patients develop T2DM. A case study reported that two siblings with AT manifested severe insulin resistance symptom [30]. Recent molecular studies have identified that cytoplasmic ATM regulates insulin-mediated signaling and glucose homeostasis by phosphorylating Akt activity and p53, which facilitates translocation of cell surface glucose transporter 4 complex and reducing Jun N-terminal kinase activity [10, 17–20]. This provides a potential molecular mechanism that underlies defective- or non-response to insulin in AT patients, leading to the development of insulin resistance and T2DM. It also suggests that there may be an association between mutations in the ATM gene and the susceptibility of T2DM.

It is known that polymorphisms in the promoter region of certain genes might regulate their expression by altering the binding sites of transcriptional factors [31]. Thus, subjects with different SNPs in the promoter region may exhibit distinct phenotypes. The present study examined the association of the SNP rs189037 in the promoter region of the ATM gene with T2DM among older adults in China. To the best of our knowledge, this is the first study to explore the relationship between ATM rs189037 polymorphism and T2DM in older adults. The results showed that the CT genotype was associated with a lower risk of T2DM than the CC or TT genotype, suggesting that CT heterozygote might be protective against T2DM. Moreover, this protective effect appeared to be more profound in male patients. Because of limited number of female subjects enrolled in our study, we could not perform the analysis in older females. Whether CT genotype has similar protective effect against T2DM in older females remains to be investigated.

One study observed that TT genotype of the ATM rs189037 polymorphism was associated with lower prevalence of diabetes mellitus in the general population [32]. The different results might be explained by the following. First, the previous study did not restrict itself to the older adult population. It has been shown that there is a significant association between the CT genotype and longevity [33]. CT genotype is likely present at higher frequencies and may exert a protective effect in old persons. Secondly, diabetic patients were limited to those with T2DM in this study, whereas the prior study enrolled patients with a history of diabetes mellitus, including type 1, gestational diabetes and other types of diabetes. As described earlier, mutations in the ATM gene typically result in T2DM [29, 30], indicating results in this study might be more reliable. However, mechanisms by which the SNP rs189037 regulates function in diabetics remains unknown. Earlier studies have demonstrated that this SNP affects the expression of ATM mRNA through differentially binding to AP-2a, an important factor that regulates transcription of the ATM gene in long-lived individuals and coronary artery disease patients [32, 33]. Whether this polymorphism also results in different expression levels of the ATM gene in T2DM patients is currently unknown and deserves further investigation.

AT patients may also be at increased risk for cardiovascular diseases. Earlier epidemiologic data indicate that the rate of ischemic heart disease-related mortality is significantly higher among heterozygous ATM carriers than that in the general population [11]. In addition, AT patients manifest higher levels of plasma cholesterol and triglycerides than normal controls [34], major risk factors for atherosclerosis. Data from a study cited earlier indicate that functional rs189037 polymorphism is significantly associated with mild coronary stenosis [32]. Animal studies have also shown that mutation in one or two ATM alleles worsen the features of metabolic syndrome, increase insulin resistance, and accelerate atherosclerosis in apoE−/− mice. Moreover, in ATM^+/−^ apoE^−/−^ mice, atherosclerosis is attenuated by transplantation with ATM+/+ bone marrow [19, 35, 36]. In the present study, the prevalence of CAD is significantly lower in participants with TT genotype than in those with CC or CT genotype (62.9% vs. 84.4% or 76.3%, respectively). However, these differences did not reach statistical significance (*P* = 0.082). The reason may be related to the small sample size. Ousset et al. [37] observed that increased reactive oxygen species levels was associated with decreased expression of ATM. It may be reasonable to speculate that higher ATM level might protect against diabetes-generated oxidative stress in coronary endothelial and reduce the increased risk of coronary stenosis induced by diabetes. In this study, we found that there was a significant difference in the rs189037 polymorphism between the T2DM patients with and without CAD, and the frequency of TT homozygote was significantly higher in T2DM patients without CAD than in those with CAD, suggesting that TT genotype might be related to the reduced risk of CAD in T2DM patients. This is consistent with the data demonstrating increased expression level of ATM associated with TT genotype reported in our previous study [32].

This study has several limitations. First, the sample size is relatively small. Second, most of the participants in our study are Han Chinese. Third, all the participants are 60 years old and above, which may have selective bias. To validate and further expand our findings, we will conduct multi-ethnic studies with larger sample sizes and no age restriction. Long-term follow-up studies of the regulatory mechanism of the SNP of the ATM gene are also warranted.

## Conclusions

In conclusion, our study suggests that the ATM rs189037 polymorphism is associated with reduced risk of T2DM in older adults in China. Specifically, CT heterozygote is associated with a lower risk of T2DM than CC or TT genotype. In addition, TT genotype might reduce the risk of CAD among older persons with T2DM.

## Additional files

Additional file 1: The raw data used in this paper. (XLS 50 kb)

Additional file 2: Supplementary tables. **Table S1.** Hardy-Weinberg equilibrium test of ATM rs189037 genotypes. **Table S2.** Genotypes distributions of ATM rs189037 polymorphism between T2DM patients with and without CAD. (DOCX 14 kb)

## Abbreviations

AT: Ataxia-telangiectasia
ATM: Ataxia telangiectasia mutated
CAD: Coronary artery disease
CIs: Confidence intervals
FPG: Fasting plasma glucose
HDL-C: High-density lipoprotein cholesterol
LDL-C: Low-density lipoprotein cholesterol
ORs: Odds ratios
PCR-RFLP: Polymerase chain reaction restriction fragment length polymorphism
SNP: Single nucleotide polymorphism
T2DM: Type 2 diabetes mellitus
TC: Total cholesterol
TG: Triglycerides
UA: Uric acid
