# Supplementary material for: Polymorphism rs189037C > T in the promoter region of the ATM gene may associate with reduced risk of T2DM in older adults in China: a case control study
Source: BMC Med Genet. 2017 Aug 14;18:84. doi: 10.1186/s12881-017-0446-z (PMC5557265; doi:10.1186/s12881-017-0446-z)
Supplement: Supplementary file 2 — Supplementary tables. Table S1. Hardy-Weinberg equilibrium test of ATM rs189037 genotypes. Table S2. Genotypes distributions of ATM rs189037 polymorphism between T2DM patients with and without CAD. (DOCX 14 kb) [file 12881_2017_446_MOESM2_ESM.docx]

**Table S1 Hardy-Weinberg equilibrium test of ATM rs189037 genotypes**

|  | T2DM group (n=80) | | |  | Control group (n=80) | | |
| --- | --- | --- | --- | --- | --- | --- | --- |
|  | CC | CT | TT |  | CC | CT | TT |
| Expected frequency, n | 24 | 40 | 16 |  | 21 | 40 | 19 |
| Actual frequency, n | 28 | 32 | 20 |  | 17 | 48 | 15 |
| χ^2^ | 3.27 | | |  | 3.20 | | |
| *P* value | 0.195 | | |  | 0.202 | | |

**Table S2 Genotypes distributions of ATM rs189037 polymorphism between T2DM patients with and without CAD**

| Genotype/allele | CAD group (n=67) | Control group (n=13) | χ^2^ | *P* value |
| --- | --- | --- | --- | --- |
| CC, n (%) | 26 (38.8) | 2 (15.4) | 11.08 | 0.004^*^ |
| CT, n (%) | 29 (43.3) | 3 (23.1) |  |  |
| TT, n (%) | 12 (17.9) | 8 (61.5) |  |  |
| C, % | 60.4 | 26.9 | 9.89 | 0.002^*^ |

* P<0.05
